# Mechanisms of T cell organotropism

**DOI:** 10.1007/s00018-016-2211-4

**Published:** 2016-04-01

**Authors:** Hongmei Fu, Eleanor Jayne Ward, Federica M. Marelli-Berg

**Affiliations:** William Harvey Research Institute, Heart Centre, Barts and the London School of Medicine and Dentistry, Queen Mary University of London, Charterhouse Square, London, EC1M 6BQ UK

**Keywords:** Homing receptors, T lymphocytes, T cell migration, Organotropism

## Abstract

Protective immunity relies upon T cell differentiation and subsequent migration to target tissues. Similarly, immune homeostasis requires the localization of regulatory T cells (Tregs) to the sites where immunity takes place. While naïve T lymphocytes recirculate predominantly in secondary lymphoid tissue, primed T cells and activated Tregs must traffic to the antigen rich non-lymphoid tissue to exert effector and regulatory responses, respectively. Following priming in draining lymph nodes, T cells acquire the ‘homing receptors’ to facilitate their access to specific tissues and organs. An additional level of topographic specificity is provided by T cells receptor recognition of antigen displayed by the endothelium. Furthermore, co-stimulatory signals (such as those induced by CD28) have been shown not only to regulate T cell activation and differentiation, but also to orchestrate the anatomy of the ensuing T cell response. We here review the molecular mechanisms supporting trafficking of both effector and regulatory T cells to specific antigen-rich tissues.

## Introduction: lymphocyte recirculation and homing

Effective immune surveillance relies on the continuous trafficking of lymphocytes through blood, lymphoid organs and non-lymphoid tissue in a process known as recirculation [1]. The physiological process by which lymphocytes leave circulation, cross endothelium, find and localize to particular tissues is known as extravasation [2]. In healthy tissue, the vascular endothelium forms a non-thrombotic, non-adhesive barrier, and is impermeable to macromolecules [3]. Upon inflammation, endothelial cells (ECs) undergo physiological and molecular changes such as the up-regulation of adhesion molecule expression. This in turn allows the exit of lymphocytes across the endothelium into the tissue without disrupting endothelial integrity.

The initial stage of lymphocyte trafficking is mostly mediated by selectins, which are responsible for lymphocyte transient tethering and rolling on the endothelium [4]. This initial contact between the circulating lymphocytes and the endothelium monolayer facilitates lymphocyte exposure to chemokines displayed on the EC surface, which induces intracellular signalling responses leading to lymphocyte integrin activation. Activated integrins bind to their ligands, such as the intercellular adhesion molecule-1 (ICAM-1), vascular cell adhesion molecule-1 (VCAM-1), and mucosal addressin cell adhesion molecule-1 (MAdCAM-1), expressed on the endothelium, resulting in the firm arrest of lymphocytes on the endothelium [5–7]. Following firm adhesion on the EC monolayer, lymphocytes eventually transmigrate into the tissue. Lymphocyte transendothelial migration can occur at the junction between apposing ECs (paracellular route) without disrupting the endothelial monolayer in a complex process known as trans-endothelial migration (TEM) or diapedesis involving the junctional adhesion molecules [8]. Alternatively, leukocytes can migrate across the EC body into the tissue known as the transcellular route [9]. Once across the endothelial barrier, migrating lymphocytes continue to move through the subendothelial matrix [8] and the extravascular tissue to the site of inflammation in response to chemotactic gradients [3]. Further details on the extravasation process can be found in a recent review by Nourshargh and colleagues [10].

A wide range of adhesion molecules and chemokines expressed on the ECs are required to bind to their counter receptors on the migrating lymphocytes, directing lymphocyte entry into the tissue [11]. The endothelium of different organs is characterized by preferential expression of combinations of these molecules, thus being endowed with the ability to selectively recruit distinct lymphocyte subsets. During priming, the local microenvironment and cellular interactions induce T lymphocytes to express a unique set of adhesion molecules and chemokine receptors (the ‘homing’ receptors) so that antigen-experienced T cells acquire the ability to interact with organ-specific ECs and migrate to distinct target tissues [12–15]. Given that recurrent infections are likely to affect the same organ, the acquisition of tissue tropism by memory T cells contributes to the effectiveness of recall responses. We here review the molecular interactions and mechanisms which underlie the ability of distinct memory T cell populations to migrate to different non-lymphoid sites where antigen is located.

## Naïve T cell homing to secondary lymphoid organs (SLOs)

Naïve and memory T cells display different surface molecules which define their functional properties and migratory patterns [16–19]. Naïve T cells have little or no ability to produce cytokines [20]. They recirculate from the blood, through secondary lymphoid organs (SLOs), into the lymphatic vessels and back to the blood [14, 21]. SLOs include peripheral lymph nodes (PLNs), spleen, gut-associated lymphoid tissue (GALT) including Peyer’s patches (PPs), and bronchus-associated lymphoid tissue [22].

Homing of naïve T cells to the PLNs requires the expression of CD62L and the chemokine receptor CCR7. CD62L binds to peripheral lymph node addressin (PNAd) expressed on the high endothelial venules (HEVs) of PLNs and mesenteric lymph nodes (MLNs) [23], while the chemokine receptor CCR7 binds to the chemokines CCL19 and CCL21 expressed by HEVs of the PLNs [24, 25]. CCL21 is also expressed in PPs [26]. Naïve T cells have also been shown to utilize CXCR4, which is the receptor for the chemokine stromal cell-derived factor 1 (SDF-1, CXCL12), to circulate through SLOs [27]. It has been shown that mineralocorticoid receptor signalling contributes to human naïve T cells migration to lymph nodes (LNs) by increasing CD62L, CCR7, and CXCR4 expression. Sleep-dependent release of endogenous aldosterone activates mineralocorticoid receptors on naïve T cells. This leads to an increase in the expression of CCR7 and CD62L, as well as of CXCR4. Central memory T cells with lymph node-homing capacity were also affected. This could explain the beneficial effect of sleep on mounting an adaptive immune response [28]. Naive T cells enter the LNs via HEVs, which are specialized postcapillary venules characterized by plump, cuboidal shapes and exclusively found in the SLOs [29, 30]. A recent study suggested that DCs sustain the entry of naive lymphocytes to LNs by modulating the phenotype of HEVs during homeostasis in adult mice. The effect of DCs on HEV is direct and requires lymphotoxin-β-receptor-dependent signalling. In the absence of DCs, HEV-mediated lymphocyte recruitment to LNs is inhibited [31].

CCR7 is also used by DCs to traffic from interstitial tissues into lymphatics and draining lymph nodes, in response to the chemokines CCL19 and CCL21. Kiermaier and colleagues showed that sialic acids on the surface of CCR7 recognize CCL21, regulating DCs trafficking to LNs. DCs deficient in polysialyltransferase ST8Sia IV, responsible for posttranslational addition of sialic acids to N- and/or O-linked glycans, fail to migrate in response to CCL21; however, their migration to CCL19 remains unchanged. Structure–function analysis also revealed that CCL21 exists in an auto-inhibited conformation, which is released upon interaction with polysialic acid addition to CCR7, allowing DCs sufficient in ST8Sia IV to respond to CCL21. DCs trafficking is abrogated in polysialyltransferase-deficient mice, manifesting as disturbed lymph node homeostasis and unresponsiveness to inflammatory stimuli [32].

Although previously thought not to require specific mechanisms for T cell recruitment, migration of naive T cells to the spleen has been recently shown to be facilitated by the angiotensin II (Ang II)/AT1 axis. Naïve T cells migrate to Ang II via the AT1 receptor. Importantly, a gradient of Ang II from peripheral blood to the spleen was measured. Higher levels of Ang II in the spleen induced upregulation of CD62L and CCR9 expression on T cells and production of CCL19 and CCL25 in the spleen, thus indirectly inducing their recruitment [33].

In humans, the majority of T cells entering the PPs are found to be naïve T cells. This process requires the interaction of integrin α_4_β_7_ on T cells and MAdCAM-1 on the mucosal vasculature [26, 34]. Consistent with this observation, human naïve T cells constitutively express low levels of α_4_β_7_ [35, 36]. Other studies subsequently showed that both CD62L and α_4_β_7_ are required in a sequential and synergistic manner to mediate naïve T cell migration to the MLNs [37]. CD62L binds to the PNAd expressed on the MLNs, which induces the initial rolling and tethering of T cells during transendothelial migration; α_4_β_7_ integrins subsequently bind to MAdCAM-1 and mediate firm adhesion and transmigration. T cell migration to the PPs is reduced by 55 % in mice that are deficient for CD62L; and mice lacking β_7_ integrin expression have been found to display a 90 % reduction in lymphocyte migration to the PPs [37]. Collectively, this evidence highlights the importance of both CD62L and α_4_β_7_ in naïve T cell homing to the GALT.

A number of studies have revealed marked differences in dwelling times of CD4^+^ and CD8^+^ naïve T cells in LNs before egressing into lymph. CD4^+^ T cells home to LNs more efficiently, traverse LNs twice as quickly. In contrast, CD8^+^ T cells enter and leave the LNs more slowly. Cell–cell contacts have been suggested to determine how long CD4^+^ and CD8^+^ T cell spend in LNs, specifically those made with DCs via major histocompatibility complex (MHC) molecules [38]. Expression of CCR7 and sphingosine-1-phosphate receptor 1 (S1PR1) has been shown to affect the time spent by T cells within LNs. CCR7 promotes retention, whereas S1PR1 expression is essential to overcome this retention signal and promote egress into the efferent lymph by interaction with its ligand sphingosine-1-phosphate (S1P) from the circulation [39–42]. CD69 is another major regulator of lymphocyte migration. CD69 directly binds S1PR1 receptor on the lymphocyte surface and mediates internalization of S1PR1, thus preventing lymphocyte egress [40, 41].

## Memory T cell homing to non-lymphoid tissue

Naïve T cells sequestered in the SLOs interact with antigens presented by DCs [43], and undergo clonal expansion and differentiation into heterogeneous antigen experienced T cells. Recently activated T cells leave the SLOs via the efferent lymphatics, enter the blood circulation through the thoracic duct and travel to the inflamed tissues. Promotion of lymphocyte egress into lymph and blood is sustained by distinct sources of S1P [44]. Tregs have been shown to regulate effector T cells (T_EFF_) migration to nonlymphoid tissue by downregulating the expression of the S1PR1 [45]. S1PR1 has also been shown to inhibit the generation of Tregs while driving T helper 1 (Th1) development via signalling through the kinase mammalian target of rapamycin (mTOR) and antagonizing the function of transforming growth factor-β (TGF-β) [46].

Primed T cells are a heterogeneous population consisting of short-lived T_EFF_, long-lived central memory T cells (T_CM_), long-lived effector memory T cells (T_EM_), and long-lived tissue resident memory T cells (T_RM_). These cells are defined by their different phenotypes, functions and migratory patterns [47, 48]. Short-lived T_EFF_ represent the majority of recently activated T cells, and are eliminated at the end of primary immune responses [19, 49, 50]. T_CM_ are able to proliferate rapidly during secondary immune responses [51]. T_CM_ express CD62L and CCR7, and share migratory patterns with naive T cells; however, unlike naïve T cells they also express inflammatory chemokine receptors which enable them to enter sites of chronic inflammation [11]. For example, T_CM_ preferentially accumulate and undergo homeostatic proliferation in the bone marrow [52, 53]. CXCR4 binding to SDF-1 play an essential role in T_CM_ homing to the bone marrow [54]. CXCR4-deficient T_CM_ exhibit defective homeostatic self-renewal, which correlates with impaired homing to the bone marrow. Upon re-challenge, however, CXCR4-deficient T_CM_ can proliferate and differentiate efficiently while self-renewing [55]. In contrast, both long-lived T_EM_ and short-lived T_EFF_ lack the expression of CD62L and CCR7. They use instead tissue-specific integrin and chemokine receptors to preferentially migrate to non-lymphoid tissues [48, 56].

Primed T cells have different degree of access to distinct tissues at steady state. Based on T cell recirculation, tissues of the body can be characterized into three classes. First, ‘permissive tissues’, such as lung parenchyma, liver, kidney, spleen and adipose tissue, are readily accessible by T_EFF_ without the need for any local inflammation or antigen [57, 58]. Second, ‘restrictive tissues’, includes skin epidermis, lung airways, vaginal epithelial layer, and salivary glands, are inaccessible by T_EFF_ at steady state. These tissues require local inflammation leading to production of inflammatory cytokines and chemokines to render ECs permissive to T_EFF_ migration [59–61]. Third, ‘effector permissive tissues’, including the gut, brain, and peritoneal cavity, are accessible by T_EFF_ during the effector phase when homing receptors are broadly expressed, but not by T_EM_ and T_CM_ [57, 62, 63].

Upon reaching the site of infection, memory T cells mount immune response, leading to pathogen clearance. Th cells regulate diverse and appropriate cellular and humoral responses to a wide range of pathogens/antigens by differentiating into a variety of subsets—Th1, Th2, Th9, Th17 and inducible Tregs (iTregs)—defined by their distinct profile of transcription factor, cytokine, function and homing receptors expression. There are various mechanisms to ensure the stability of different T cell subsets, such as expression of genes turned on by transcription factors, repression of genes conferring alternative fates, epigenetic modifications and microRNA expression. However, there are many examples of flexible expression of cytokines, transcription factors and homing receptors by established Th cells [64–66]. For example, Th17 cells can simultaneously produce IL-17 and IFN- γ [67], or IL-17 and IL-4 [68].

Migration of Th cells to peripheral sites of inflammation is essential for execution of their effector function. Different Th cell subsets may use different adhesion molecules and chemokine receptors to traffic during inflammation. It has been reported that binding of T cell immunoglobulin and mucin domain 1 (TIM-1) to P-selectin mediates tethering and rolling of Th1 and Th17, but not Th2 and Tregs [69]. The chemokine receptors CXCR3, CXCR6 and CCR5 are preferentially expressed on Th1 cells [70, 71], whereas CCR3, CCR4 and CCR8, along with the prostaglandin D_2_ receptor CRTH2 (chemoattractant receptor homologous molecule on Th2 cells), are expressed on Th2 cells [71–73]. More recently, CCR6 and CCR4 have been shown to characterize the Th17 subset, although there is also evidence that Th17 cells may express CCR2 and CCR9 [74–77]. In addition, the same Th cell lineage may use different receptors to migrate to inflammation sites depending on disease models. For example, Th9 cells express functional chemokine receptors CCR3, CCR6, and CXCR3 for the recruitment to disparate inflammatory sites; however, during allergic inflammation, Th9 cells preferentially use CCR3 and CCR6, not CXCR3; finally, Th9 homing to the central nervous system (CNS) during experimental autoimmune encephalomyelitis (EAE) involves CXCR3 and CCR6 but not CCR3 [78].

At the end of immune reaction, the tissue microenvironment provides instructive signals for some T_EFF_ to express molecules that enable long-term residency and survival to become T_RM_. T_RM_ does not readily circulate and comprises a major component of lymphocyte populations in diverse peripheral tissue sites, including mucosal tissues, barrier surfaces, and other lymphoid and non-lymphoid sites in humans and mice [79–84]. Functionally, T_RM_ provide an immediate in situ immune response to infection due to their localization in barrier tissues, and also contain relatively high frequencies of T cell clones specific for pathogens as T_RM_ are likely derived from clonally expanded T_EFF_ responding to an infection [85–87]. Phenotypically, T_RM_ are distinguished from circulating T_EM_ based on upregulated expression of CD69 by both CD4 and CD8 T_RM_, and CD103 by CD8 T_RM_ [83, 84]. E-cadherin, the ligand for CD103, is expressed on epithelial cells. It is possible that interactions between E-cadherin and CD103 contribute to maintaining T_RM_ in peripheral tissues. TGF-β, a cytokine secreted by various skin and mucosal epithelial cells [88, 89], stimulates CD8 T cells to express the integrin CD103 [90]. Antigen-stimulated CD69 expression by T_RM_ inhibit the egress receptor S1PR1 by binding to the transmembrane region of S1PR1 and promotes its degradation [91]. S1PR1 responds to concentrations of S1P, which is scarce in the tissue but highly abundant in blood and lymph [42]. S1PR1 expression requires transcription factor Krüppel-like factor 2 (KLF2), which is downregulated once T_EM_ seed the peripheral tissue [92]. Downregulation of KLF2 also decreases the expression of CCR7, which is another egress receptor on tissue lymphocytes required for their migration into the draining LNs [93, 94].

T_RM_ have been reported to survive in memory lymphocyte clusters (MLCs), which are formed within the parenchyma of the tissue after a local infection or immunization. MLCs contain CD4 T_RM_ and CD8 T_RM_ as well as antigen presenting cells (APCs) that hold the cluster together [95]. MLCs appear to contain a depot of low levels of antigenic peptides that provide antigenic stimuli for T_RM_ retention [63, 96]. How T_RM_ are released from MLCs to migrate toward the infected cells remains to be determined.

Like other memory T cells, T_RM_ survival depends on the delivery of signals such as those mediated by IL-15 [93]. Recently Adachi and colleagues demonstrated that the epidermotropism of T_RM_ is supported by the hair follicle-derived cytokines IL-15 and IL-7. They showed that skin CD4 and CD8 T_RM_ reside predominantly within the hair follicle epithelium of the unperturbed epidermis. Hair follicle expression of IL-15 was required for CD8 T_RM_ cells, and IL-7 for both CD8 and CD4 T_RM_ to exert epidermotropism. A lack hair follicle-derived cytokines led to the failure of both CD4 and CD8 T_RM_ to persist in the skin, leading to impaired hapten-induced contact hypersensitivity responses [97]. A recent study by Mackay and colleagues demonstrated that the T-box transcription factors (TFs) Eomes and T-bet combine to control the expression of TGF-β and IL-15, which are pivotal for CD8^+^CD103^+^ T_RM_ development and survival. They showed that extinguishment of Eomes was necessary for CD8^+^CD103^+^ T_RM_ development, and residual T-bet expression maintained cell surface IL-15 receptor β-chain (CD122) expression necessary for IL-15-induced survival signals [98].

Another study by Hondowicz and colleagues revealed that interleukin-2 (IL-2) signaling was required for the formation of lung-resident memory Th2 cells, drivers of lung allergic responses. They found that significantly greater numbers of CD25-sufficient than CD25-deficient allergen-specific Th2 cells were found in the lungs after intranasal administration of allergen. They further showed that IL-2 regulates a broad migrational program of lymphocyte retention and migration, regulating the early residency of allergen-specific CD4^+^ T cells in the lung [99].

Memory T cells can also leave parenchymal tissue and return to the circulation. The mechanisms leading to the release of memory T cells from parenchymal tissue into the circulation have been only partially characterised. The chemokine receptor CCR7 has been proposed to guide T cell exit from peripheral tissues and entry into afferent lymphatics [100]. However, a conflicting report suggests that CCR7 plays a discernible role in the trafficking of memory CD4^+^ T cells to the LN, either directly from the blood or from peripheral tissues such a skin [62]. A study has reported that murine T_EM_ and T_EFF_ can also be recruited to the reactive LNs in a CCR7-independent pathway via the chemokine receptor CXCR3 to kill antigen-presenting DCs thus preventing excessive T cell stimulation [101]. CXCR3 is upregulated on both Th1 and CD8^+^ T cells upon activation and maintained on these T cell subsets throughout the effector and memory phase [102].

After exit from the tissue, memory T cells may continue recirculating in the blood or localize in ‘reservoir’ niches: memory T cells tend to preferentially reside in the spleen in chronic inflammation with antigen persistence; alternatively they localize to the bone marrow if the infectious/inflammatory process is acute but transient [48, 103, 104].

### Memory T cell homing to the skin

Effector/memory T cells trafficking to skin depends on the expression of cutaneous lymphocyte-associated antigen (CLA), CCR4 and CCR10 [105]. It has been demonstrated that human memory T cells require the expression of CLA to traffic to the skin by binding to E-selectin, which is constitutively expressed at a low level by ECs in the skin and is upregulated upon cutaneous inflammation [106, 107]. Human skin-infiltrating lymphocytes also express CCR4, the chemokine receptor for CCL17 (also known as the thymus and activation-regulated chemokine, TARC) displayed on the skin post-capillary venules [108]. CCL17 is produced by dendritic cells (DC), endothelial cells, keratinocytes and fibroblasts [109]. Further studies revealed that CCL27 (the cutaneous T cell-attracting chemokine, CTACK), expressed by skin keratinocytes, also contributes to memory T cells recruitment to the skin by binding to its receptor CCR10 [110]. Other key molecules participating in memory T cell homing to the skin include the integrin α_4_β_1_ (VLA-4), which binds to VCAM-1 expressed by the endothelium [36]. T cells expressing high levels of α_4_β_1_ preferentially home to the systemic non-mucosal sites including the skin, CNS and bone marrow [111].

The requirement and redundancy of individual chemokine receptors in T cell homing to the skin is still controversial. Marked reduction of dinitrofluorobenzene-induced allergic contact dermatitis (ACD) was observed following neutralization of CCL27–CCR10 interactions in Balb/c mice [112] and in CCL17-deficient mice [113]. However, Reiss and colleagues reported that simultaneous inhibition of CCR4 and CCR10 was required to block lymphocyte recruitment to reduce dinitrofluorobenzene-induced ACD [108]. Similarly, Mirshahpanah et al. demonstrated that only combination therapy of neutralizing antibodies to CCL17, CCL27 and CCL22 resulted in oedema inhibition using three different ACD models [114]. Differences in the relative contribution requirements for individual chemokine receptors in T cell homing to the skin may be in part explained by different genetic background and experimental models used in these studies.

The skin tissue microenvironment provides instructive signals for some recruited effector/memory T cells to express molecules that enable long-term residency and survival to become skin T_RM_. It has recently emerged that skin T_RM_ are crucial for protection not only against the antigenic pathogen but also against antigenically unrelated viruses and bacteria. Activated skin T_RM_ can profoundly alter the local tissue environment by inducing a number of broadly active antiviral and antibacterial genes [115]. The mouse skin has been shown to harbour the so-called dendritic epidermal T cells (DETCs), a unique, highly specialized lymphocyte subset characterized by its dendritic shape [116–118]. DETCs participate in various activities including wound healing and epidermal barrier function [117–119]. Embryonic trafficking of DETCs to the epidermal skin requires G protein-coupled receptor 15 (GPR15) [120]. DETCs in GPR15^−/−^ mice remain low at birth, but later participation of vascular E-selectin, CCR4 [105] and CCR10 [121] allows DETCs to reach near normal levels in adult skin.

### Memory T cell homing to the intestine

Effector and memory T cells require the interaction of integrin α_4_β_7_ and MAdCAM-1 to migrate to the effector site of the gut mucosa—the lamina propria (LP) [34, 122]. MAdCAM-1 is constitutively expressed on the postcapillary venules of intestinal LP and MLNs, the HEVs of the PPs [12] and the endothelial venules of both small intestine and colon [123]. The ability of α_4_β_7_ expressing cells to bind to MAdCAM-1 is influenced by the level of α_4_β_7_ expression and its functional state [35]. Human peripheral blood CD4^+^, CD8^+^ T cells have heterogeneous expression of α_4_β_7_ [35, 124]. The level of α_4_β_7_ expression is much higher on memory cells than naïve cells [35, 36]. Other than its crucial function in mediating lymphocyte homing to the gut, studies in mice have also revealed that α_4_β_7_ plays a critical role in the formation of GALT [125]. Mice lacking MAdCAM-1 have a disordered architecture in their PPs and MLNs [126]. α_4_β_7_-dependent gut trafficking is essential but not sufficient to induce intestinal inflammation. Studies demonstrated that differential ability for α_4_β_7_^+^ T cells to become colitogenic may be linked to their antigen reactivity, and the reactivity was enhanced by the presence of γδ T cells [127].

In addition to its role in adaptive immunity, α_4_β_7_ is also required for innate immune cells to migrate to intestine. Villablanca et al. showed that β_7_ integrins are required for bone marrow progenitors to reconstitute the small intestine mononuclear phagocyte compartment including retinoic acid (RA)-producing DCs in mLNs. Lack of β_7_ integrins in the innate immune compartment markedly accelerated T cell-mediated colitis due to decreased ability to induce Tregs and IL-10-producing T cells [128].

Interestingly, interaction of α_4_β_7_ with MAdCAM-1 has been proposed to contribute to immune tolerance in other organs by selective sequestration of pro-inflammatory T cells in the intestine. Berer and colleagues found that the pro-inflammatory, myelin-reactive Th17 cells were enriched in the intestine of EAE-susceptible mice, but failed to reach the peripheral immune organs, resulting in the absence of spontaneous EAE. The release of intestine-sequestered Th17 cells via blocking of α_4_β_7_-mediated adhesion to the intestine by treating mice with α_4_β_7_-specific monoclonal antibodies worsened EAE [129]. Similarly, treatment of EAE mice with IL-4 increased retinaldehyde dehydrogenase (RALDH) activity in DCs, which enhanced RA production, induced expression of α_4_β_7_ and CCR9 on CD4^+^ T cells and deviated their migration from the dLNs-CNS route to the mLNs-gut thus improving EAE outcome without overt colitis [130].

Upon arrival in the intestinal LP, some T cells further migrate to the gut epithelium to become intraepithelial lymphocytes (IELs) in a process dependent on the interaction of chemokine receptor CCR9 and its ligand CCL25 [131–133]. In mice, the CCR9–CCL25 axis has been shown to play a key role in the homing of antigen-specific CD8^+^ T lymphocytes to the small intestine LP [134], but less so for CD4^+^ effector T cells [135]. CCR9 is expressed by thymic T cells, α_4_β_7_^+^ LP T cells and IELs in the small intestine in humans and mice [132, 133, 136]. CCL25 is expressed in the thymus and the small intestinal glands and crypts [137]. CCL25 has also been found to be expressed by gut endothelium and epithelial cells in mice [138]. Within the murine small intestine, CCL25 is found at a higher concentration at the proximal segments compared to the distal segments [138]. In humans, CCL25 is abundantly expressed in various regions of the small intestine (jejunum and the ileum), but not in the colon or the caecum [132, 139]. In line with these observations, CCR9–CCL25 interactions are required for memory T cell homing to the small intestine, while they do not appear to be essential for T cell migration to the colon in humans or mice [132, 140]. Blockade of CCL25 had no effect on murine T cell adhesion in the colon in both non-inflamed and inflamed conditions, while it significantly inhibited T cell adhesion in the small intestine [137, 141]. Consistent with these findings, over 90 % of both CD4^+^ and CD8^+^ T cells isolated from the human small intestine express CCR9, in contrast only 25 % of colonic CD4^+^ and CD8^+^ T cells are CCR9 positive [132, 139]. Only a small percentage of total circulating CD4^+^ and CD8^+^ T cells co-express α_4_β_7_ and CCR9 in human peripheral blood, further enforcing the concept that T cell homing to the small and large intestine may not be achieved via the same pathways [133]. Instead, CCL25 may be responsible for compartmentalizing the mucosal immune responses within the gut [132, 136].

Optimal expression of α_4_β_7_ and CCR9, particularly by CD4^+^ T cells, has been found to be regulated by basic leucine zipper transcription factor (BATF), an AP-1 protein family factor. Wand and colleagues showed that BATF is required for CD4^+^ T cells to up-regulate the gut-homing receptors in response to RA upon antigen priming and migrate into and populate the intestine. BATF deficient CD4^+^ T cells failed to up-regulate the expression of CCR9 and α_4_β_7_ to wild type levels in response to RA, and are ineffective in migration into the intestine [142].

The concept that immune surveillance of the intestine is dependent on a large cohort of recirculating α_4_β_7_^high^ memory T cells has recently been challenged by a seminal study showing that the expression of α_4_β_7_ is lost by the majority of circulating memory T cells—including those activated in the GALT—despite the maintenance of protective immunity [62]. In addition, loss of α_4_β_7_ and CCR9-mediated interactions by genetic deletion does not prevent gut immunity from taking place [140, 143], suggesting the existence of redundant mechanisms of T cell migration to the intestine.

A few other molecules also play a non-redundant role in intestinal homeostasis. Soluble lymphotoxin α produced by RORγt^+^ innate lymphoid cells induce GALT development. Mice deficient in soluble lymphotoxin α lacked GALT such as PPs, abrogated IgA production in the gut and altered microbiota composition via regulation of T cell homing to the gut [144] as T cell-dependent regulation of IgA production takes place mainly in PPs and requires the formation of germinal centres and the interaction of B cells with follicular Th cells [145]. Additionally, GPR18 has been shown to promote the maintenance of small intestinal IELs. Mice lacking this orphan receptor have reduced numbers of the CD8αα, CD8αβ, and γδT IELs [146]. GPR15 was also involved in the homing of T cells, particularly Forkhead Box P3 (FoxP3)^+^ Tregs, to the large intestine LP. GPR15-deficient mice were prone to develop more severe large intestine inflammation. Unlike α_4_β_7_ and CCR9, GPR15 expression was not modulated by RA, but by gut microbiota and TGF-β–1 [147].

### Memory T cell homing to other mucosal sites

Unlike well-defined T cell homing to the gut, the molecular pathways regulating the migration of T cells to other mucosal sites are not fully defined. A few molecular mechanisms have been suggested to contribute to T cells homing to the airway. Danilova and colleagues suggested that CCR3 is involved in the steady-state trafficking of CD4^+^ T cells to the human upper airway mucosa [148], as CCR3 ligand CCL28 is preferentially expressed in various mucosal tissues of the upper aerodigestive tract [149, 150]. Another study proposed that CCR4 contributes to T cell lung homing imprinting. It was found that lung DCs induce the expression of CCR4 on T cells. Lung DCs-activated T cells traffic more efficiently into the lung and protect against influenza more effectively compared with T cells activated by DCs from other tissues [151]. Lim and colleagues suggested that CXCR4 plays a role in CD8^+^ T cell migration to airway tissues. They demonstrated that early recruited neutrophils into influenza-infected trachea deposit long-lasting SDF-1-containing trails, which provide both chemotactic and haptotactic cues for efficient CXCR4 expressing CD8^+^ T cell migration and effector functions in influenza-infected tissues [152].

Molecular mechanisms regulating the migration of T cells to the female genital tract have also been studied. Shannon and colleagues showed that asymptomatic herpes simplex virus (HSV)-2 infection was associated with systemic T cell immune activation and a dramatic increase in their expression of the mucosal homing α_4_β_7_ and CCR5, which directly correlated with the number of activated and CCR5-expressing human immunodeficiency virus (HIV)-susceptible CD4^+^ T cell subsets in the cervix [153]. This may explain why asymptomatic HSV-2 infection is associated with a threefold increase in HIV acquisition risk [154]. In contrast, another study showed that integrin α_4_β_1_, but not α_4_β_7_, contributes to CD4^+^ T cell trafficking to the uterus. It was demonstrated that *C. trachomatis* infection of the upper genital tract results in recruitment of chlamydia-specific CD4^+^ T cells robustly expressing the integrin α_4_β_1_. Blocking or deleting α_4_β_1_, but not α_4_β_7_, on pathogen-specific CD4^+^ T cells results in the impairment of trafficking to the uterus and high bacterial load [155]. Unique challenges posed by HIV or other sexually transmitted infections such as HSV require further research on memory lymphocytes generation against HIV or HSV with mucosal tissue tropism to design effective T cell-based vaccines.

### Memory T cell homing to the liver and the heart

T cell homing to the liver has received much attention in recent years, and a number of molecular mediators of T cell localization to hepatic tissue have been identified. Studies in experimental models of liver inflammation have indicated that Th1 cells may use VLA-4 to traffic to liver, whilst Th2 cells may use a presently uncharacterized ligand for endothelial vascular adhesion protein-1 (VAP-1), which is constitutively expressed on hepatic venules and liver sinusoids [156]. Other reports suggested the involvement of the hyaluronan receptor CD44 in lymphocyte homing to liver [157]. CCR5 has also been suggested as a mediator of recruitment of T cells in the liver during acute inflammation as well as during numerous autoimmune diseases, including multiple sclerosis, rheumatoid arthritis and type 1 diabetes [158]. First, CCR5 is preferentially expressed on Th1 cells, and Th1 cell-mediated immune responses play a critical role in hepatocyte damage induced by autoimmunity and viral infections [159, 160]. Second, it was found that some CCR5 antagonists might induce profound hepatotoxicity during clinical trials [158]. Third, CCR5 blockade/deficiency is associated with significant increase in tissue levels of the CCR5 ligand CCL5 [161, 162], which can promote enhanced influx of leukocytes (including T cells) by binding to its alternative receptor, CCR1, expressed on circulating leukocytes [161, 163].

Besides homing to the skin and liver, it has been challenging to identify unique tissue-homing signatures to other solid organs including the heart. It has been shown previously that the chemokine receptors CCR4 [164] and CXCR3 [165] are contributing to T cell accumulation during heart transplant rejection. Recently we have uncovered a molecular mechanism of induction of T cell cardiotropism. We found that engagement of the hepatocyte growth factor (HGF) receptor c-Met by heart-produced HGF during priming in the LNs instructs T cell cardiotropism, which was associated with a specialized homing “signature” (c-Met^+^CCR4^+^CXCR3^+^). HGF is expressed by healthy heart tissue and transported to local draining LNs. Inside heart draining LNs, HGF bind to c-Met on naive T cells, inducing higher expression of c-Met itself and of the chemokine receptors CCR4 and CXCR3. C-Met triggering was sufficient to support cardiotropic T cell recirculation, while CCR4 and CXCR3 sustained recruitment during heart inflammation. In steady state conditions, engagement of cMet induces autocrine release of beta chemokines, which favour T cell recruitment via their receptor CCR5. Under inflammatory conditions, cardiac tissue releases higher levels of the HGF and chemokines CXCL10 and CCL4, which facilitate HGF-primed T cells recruitment to the heart [166].

### Mechanisms of homing receptor acquisition

The ability of local microenvironment to imprint T lymphocytes with a specific set of homing receptors has long been recognized. Tissue-associated DCs appear to be capable of imprinting the tropism of a T cell during the priming phase. It was first demonstrated in mice that only DCs isolated from the MLNs and PPs preferentially up-regulated gut-homing receptors α_4_β_7_ and CCR9 expression when activating naïve T cells [134, 167, 168]. In contrast, T cells activated in the cutaneous secondary lymphoid tissue expressed skin-homing receptors such as P-selectin glycoprotein ligand-1 (PSGL-1; CD162) [168, 169]. The mutually exclusive sets of skin and gut-homing receptors expressed by T cells commit them to either destination [36, 133].

More recent studies have shed light on the molecular mechanisms of ‘local’ imprinting. While tissue-associated DCs appear to be capable of imprinting the tropism of a T cell during the priming phase, additional signals within the tissue microenvironment may be required to imprint and maintain a particular homing program. A study by Iwata and colleagues highlighted the key role of the vitamin A (VA) metabolite RA in the imprinting of gut homing specificity on T cells [170]. VA enters the body exclusively through diet [171], absorbed and processed primarily in the small intestine [172]. VA is inactive and requires enzymatic processing to become activated [173]. It is first converted to retinal catalysed by ubiquitous alcohol dehydrogenases (ADH), and then to RA catalysed by cell specific RALDH [170, 174–176]. DCs in GALT express RALDH, which metabolise VA to RA [170, 171]. RA along with antigens is presented by DCs to naïve T cells during priming. RA binds to its receptors—RAR and RXR expressed on naïve T cells, which then up-regulate the expression of α_4_β_7_ and CCR9 [170, 171, 177–180]. CD103^+^ DCs express higher levels of enzymes required for VA metabolism compared to CD103^−^ DCs, represent the major migratory DC population in the intestinal LP and are believed to be most effective at generating α_4_β_7_^+^ CCR9^+^ CD8^+^ T cells compared to DCs from MLNs or PPs [181]. However, The MLNs are believed to serve as the primary site where imprinting of gut homing specificity occurs as antigen-loaded DCs are carried into the MLNs via draining lymphatics [182–186].

DCs can also internalize exogenous RA produced by other cells in the gut such as LP macrophages [176], MLN stromal cells [187] and gut epithelial cells [188]. However, the contribution of RA from sources other than DCs towards the imprinting process is not fully established [189]. RA can also be produced by other DC populations, particularly in DCs located at the environmental interfaces, such as the skin, the lungs, and the corresponding draining LNs [190, 191]. Compared to DCs from GALT, DCs from the environmental interfaces are shown to be less effective at converting VA to RA, and are therefore less effective at inducing α_4_β_7_ and CCR9 expression on T cells [170].

In addition to the role of producing and presenting RA to T cells, DCs also express the RA receptors and are able to respond directly to RA [192]. RA can modulate monocyte-derived DCs towards a mucosal-type DCs, which produce IL-6 and TGF-β and has capacity to induce gut-homing receptors on T cells [193]. RA can also influence the migratory properties of DCs. RA induces expression of the matrix metalloproteinases (MMP)-9 and MMP-14 [194, 195]. MMP-9 and MMP-14 are involved in DC migration [196, 197]. RA promotes the migration of DCs in vivo toward draining LNs in a murine model of cancer [194]. Similar mechanisms involving VA metabolism are also used to instruct Tregs and B cells to become gut-tropic [180, 198–200].

Apart from inducing a gut-homing phenotype, RA also prevents the upregulation of skin-homing receptors by T cells [170]. It has been suggested that the acquisition of a skin-homing phenotype might be the default pathway for T cell activation in the absence of RA or when RAR signalling is blocked [201]. For example, a distinct inflammatory Th2 with homing receptors for skin and inflammatory sites was induced by DCs from MLNs in VA-deficient mice. The MLN-DC subset from VA-sufficient mice induced a similar T cell subset with homing receptors for skin and inflammatory sites in the presence of RAR antagonists. RA inhibited this induction [202].

Like the role of VA in the gut-homing imprinting, vitamin D_3_ has been implicated in the induction of a skin-homing phenotype in T cells. Skin-resident DCs have been shown to process vitamin D_3_, the inactive pro-hormone naturally generated in the skin by exposure to the sun, to 1,25(OH)(2)D(3), the active form of vitamin D_3_. 1,25(OH)(2)D(3) signals have been shown to induce CCR10 expression, which promote T cell trafficking to and/or retention in the skin epidermis via binding to its ligand CCL27 secreted by keratinocytes [173]. In contrast, 1,25(OH)(2)D(3) suppresses the expression of the gut-homing receptors α_4_β_7_ and CCR9. Skin stromal cell-derived prostaglandin E2 can also decrease gut-homing CCR9 expression upon T cell priming via inhibiting RALDH expression in mouse and human DCs. It was found that prostaglandin E2 stimulated the expression of the inducible cyclic AMP early repressor, which appears to directly inhibit RALDH expression in DCs [203].

Recent findings have provided strong evidence that soluble factors produced in selected tissues and drained in the local lymphatic stations contribute to the topographic imprinting of T cells during activation. It is known that tissue-derived small molecules can be directly delivered to draining LNs by anatomically defined conduits [204]. Some of these molecules are produced in a tissue-specific manner and can therefore define the topographic identity of the tissue where they are generated and possibly contribute to T cell homing imprinting. For example, skin-derived soluble factors have been shown to induce the skin homing receptor CCR8 in T cells [205]. We have reported recently that HGF produced by the myocardium is transported to the heart-draining LN either passively or bound to DCs [166]. Binding of HGF to its receptor c-Met on T cells induce intracellular signalling cascade involving Akt and STAT3 engagement, leading to increased expression of CCR4 and CXCR3 and secretion of several chemokines, including CCL3, CCL4, CCL5, CCL17, and CCL22. This mechanism represents an autocrine loop, by which T cells—once primed by HGF—stimulate both receptors and their ligands to re-direct T cells to heart tissue.

**Fig. 1:**
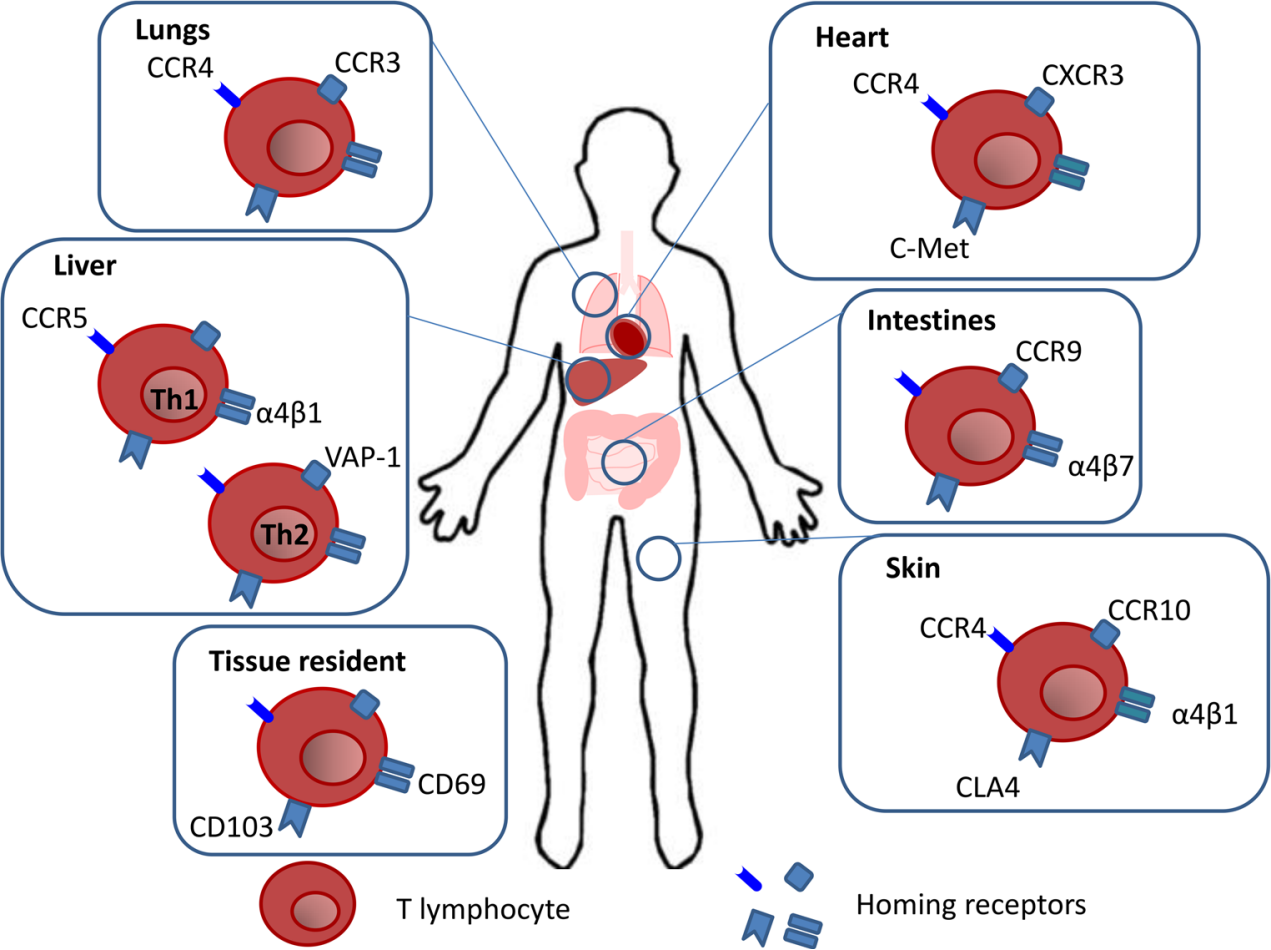
Memory T cell tissue homing. Memory T lymphocytes acquire the expression of homing receptors, imprinted during priming in the draining LNs, which enable their migration to specific tissues. T cells trafficking to the heart occurs due to the expression of C-met which binds to its ligand HGF released by the heart. This binding induces the upregulation of CCR4 and CXCR3. The interaction of α4β7 and CCR9 with their respective ligands MAdCAM-1 and CCL25 results in gut specific homing. Migration into the skin occurs due to the expression of CCR4, CCR10, α4β1 and CLA which interact with their respective ligands CCL17, CCL27, VCAM-1 and E-selectin on the endothelium. Th1 cell homing to the liver is been reported to use VLA-4 and CCR5 whereas Th2 cells use VAP-1 which is expressed on hepatic venules and liver sinusoids. CCL3 which binds to CCR3 and CCR4 have been shown to contribute to imprinting a lung homing signature. At the end of an immune reaction some T lymphocytes become tissue resident following upregulation of CD69 and CD103

A summary of the homing receptors used by memory T cells to migrate to different tissues is provided in Fig. 1.

### T cell receptor: the ultimate addressin?

Primed T cells must patrol very large areas to locate antigen-rich tissue to exert their function. Together with the acquisition of homing receptors during activation and differentiation, additional antigen-driven mechanisms have been proposed to orchestrate the targeted delivery of memory T cells to antigen-rich tissues. Several mechanisms could contribute to the accumulation of antigen-specific T cells at antigen-bearing sites: (a) a direct effect of antigen recognition on the recruitment of T cells; (b) the retention of antigen-specific T cells; (c) local proliferation of antigen-specific cells.

T cell receptor (TCR) recognition of antigen/MHC complexes presented by the endothelium has been reported both in vitro and in vivo to contribute directly to the recruitment of antigen-specific T cells. In vitro, cognate recognition of B7-deficient human and murine ECs was shown to enhance T cell trans-endothelial migration without inducing unresponsiveness [206–208]. In vivo, antigen presentation by the endothelium has also been shown to sustain the recruitment of specific T cells to antigenic sites. For instance, islet-specific homing by insulin-specific CD8^+^ T cells was abrogated in mice lacking MHC class I (MHC I) expression, or in mice displaying impaired insulin peptide presentation by local endothelium due to lack of insulin secretion [209]. Similarly, up-regulation of male antigen HY presentation by local vessels led to peritoneal recruitment of HY specific CD8^+^ lymphocytes T cells in male, but not female mice [210]. Indirect evidence implicating the requirement of MHC I molecule upregulation for T cell localization to antigen-rich non-lymphoid tissue was first provided by studies showing that homing of insulin-specific CD8^+^ T cells to the islets during the onset of autoimmune diabetes in non-obese diabetic (NOD) mice in vivo was impaired in IFN-γ-deficient NOD mice [211]. In particular, T cell diapedesis was significantly diminished. This effect was reversible by treatment of the animals with recombinant IFN-γ. MHC class II molecule expression by microvascular endothelium in the CNS precedes and is required for the formation of T cell infiltrates in EAE in guinea pigs [212]. Presentation of cognate antigen by either ECs or bone marrow-derived APCs that extend into the capillary lumen was sufficient for T cell migration into graft tissues [213]. Intravital microscopy revealed that antigen presentation by the endothelium selectively enhances T cell diapedesis into the tissue without affecting rolling and adhesion.

TCR signals can also exert indirect effects on T cell migration. The identification of a crosstalk between TCR- and chemokine receptor-mediated signalling indicates that TCR-induced signals might also affect T cell responses to chemokines. Both TCR signalling and chemokine receptor signalling trigger downstream integrin activation, e.g, Lymphocyte function-associated antigen (LFA)-1 and VLA-4 [214, 215]. Moreover, signal transduction induced by CXCR4 in human T cells requires the zeta-associated protein 70 (ZAP-70), a key element in TCR signalling [216]. In addition, chemokine receptor expression is regulated by T cell activation [217]. Chemokines contribute to antigen-specific T cells homing following cognate recognition of the endothelium in vivo [209, 218].

The relative contribution of antigen-specific and non-antigen-specific signals to memory T cell recruitment is likely to be determined by the intensity of the inflammatory response. It is possible that TCR-mediated primed T cell localization to antigenic sites may be essential to ensure efficient, rapid memory responses in the event of limited inflammatory signals. For example, insulin-specific T cells are efficiently recruited to pancreatic islets of various mouse strains that are free of pre-existent inflammation [209]. In contrast, severe inflammation or a pre-existing large antigen-specific T cell repertoire (for example during direct alloresponses) may override the requirement for selective antigen-dependent T cell recruitment [219].

### Co-stimulatory molecules regulate T cell migration

Co-stimulatory signals such as those mediated by CD28 delivered to T cells in conjunction with TCR engagement are required to sustain T cell division, differentiation and survival [220]. Negative co-stimulators (such as CTLA-4) counteract these effects thus promoting homeostatic mechanisms and preventing autoimmunity. These co-stimulators have also been shown to regulate adhesion molecules activity and cytoskeletal rearrangement in vitro [221–223]. In vivo, CD28-mediated signals are required for primed T cells to leave lymphoid tissue following priming and promote the localization of T cells to target tissue. A prominent feature of CD28-deficient immune responses is the inefficient localization of primed T cells to non-lymphoid antigenic site [224–227]. TCR-transgenic T cells carrying a mutation in the cytoplasmic tail of CD28 (CD28^Y170F^) that abrogates phosphatidylinositol-3′-kinase (PI3K) recruitment without leading to defects in clonal expansion failed to localize to target tissue following priming [227, 228].

The mechanism by which CD28 facilitates migration of primed T cells to non-lymphoid tissue is unclear. CD28 does not appear to directly mediate adhesion [229], but may favour primed T cell access to non-lymphoid tissue by inducing integrin mediated-adhesion. Additional mechanisms, such as transcriptional regulation of chemokine receptor expression by CD28, are likely to be involved [227, 230]. Despite sharing adhesion-inducing and pro-migratory properties in vitro [231], CTLA-4-mediated signals lead to effects antagonistic to those induced by CD28 on T cell migration in vivo. Tissue infiltration by murine T cells was abrogated by CTLA-4 ligation [227].

In addition to CD28 and CTLA-4, a number of other costimulatory molecules have been implicated in the regulation of T cell migration. For example, OX40/OX40 ligand interaction has been shown to be required for T cell migration to germinal centres [230], and the inducible costimulatory molecule (ICOS) regulates human memory T cell migration through tumour necrosis factor (TNF)-α-treated ECs [232].

### Mechanisms of TCR- and costimulation-driven T cell trafficking: the central role of PI3K signals

PI3K signaling mediates not only events downstream of TCR and CD28 [233, 234] leading to T cell division and differentiation, but also events leading to T cells migration. It has been shown that both TCR- and CD28-driven T cell migration rely upon PI3K p110δ activity [227, 235–238]. Studies using T cells from mice expressing a catalytically inactive p110δ isoform or treated with selective p110δ inhibitor, revealed an essential role for this molecule in TCR-dependent localization of both CD4^+^ and CD8^+^ T cells in an antigen-specific transplantation model [235, 239]. Recent evidence also strongly correlates CD28-induced migration with PI3K (likely p110δ) signalling. TCR-transgenic mice carrying an ovalbumin-specific T cell receptor (OT-II) and a mutation in the cytoplasmic tail of CD28 that abrogates class I PI3K recruitment without leading to defects in clonal expansion (CD28^Y170F^) were generated to allow discrimination of conventional co-stimulation-driven clonal-expansion from their ability to infiltrate antigenic tissue (OT-II/CD28^Y170F^). OT-II and OT-II/CD28^Y170F^ naïve T cells proliferated equivalently following immunization with antigenic peptide. Unlike OT-II cells, However, OT-II/CD28^Y170F^ CD8^+^ memory T cells failed to localize to target tissue upon antigen challenge [228].

A few mechanisms have been suggested to account for the role of TCR- and CD28-induced PI3K signaling in memory T cell migration into target tissue. Naïve T cells express CD62L, CCR7 and S1PR1, which facilitate their trafficking to SLOs. Following TCR and CD28 engagement, the PI3K-Akt-mTOR axis promotes the transcriptional downregulation of CD62L, CCR7 and S1PR1 via the inhibition of the transcription factor KLF2, leading to T cells egress from draining LNs and traffic into non-lymphoid tissues [93, 94, 240, 241]. PI3K-Akt signaling has also been implicated in F-actin polymerization and myosin assembly [242–245]. Accordingly, PI3K(s) contribute to several aspects of the migratory machinery, including gradient sensing, signal amplification, actin reorganization and hence cell motility [246–248].

Activation of PI3K is also a robust signaling event shared by most homoeostatic and inflammatory chemokine receptors expressed on T cells [249]. There has been interest in exploring the role of PI3K-dependent signaling in the T cell migratory responses after chemokine stimulation. Pharmacological and genetic approaches have been employed to assess the contribution of individual class I PI3K isoforms to the T-lymphocyte’s migratory response to several chemokines. In contrast to TCR or CD28-induced migration, PI3K inhibitors were found to have little effect on chemokine induced T cell migration both in vitro and in vivo. T-lymphocyte arrest and adhesion to HEVs in exteriorized PP [250] or transendothelial migration in laminar-flow chambers [251] in response to either CXCR4 and/or CCR7 ligation is unaffected by PI3K inhibitors. There was no defect in the p110δ mutant mice of either normal constitutive trafficking or migratory response to chemokines. Further studies revealed that responses to chemokines in T cells require a largely PI3K-independent activation of the small GTPase DOCK2, a mammalian homolog of *Caenorhabditis elegans* CED-5 and *Drosophila melanogaster* Myoblast City [252].

## Regulatory T cell (Treg) trafficking

Tregs were first identified in mice by Sakaguchi and co-workers in 1995 as a subset of CD4^+^ T cells with a constitutive expression of IL-2 receptor alpha-chain CD25 [253]. Tregs are characterized by constitutive expression of the transcription factor FoxP3, which confers on them potent suppressive activity [254, 255]. Tregs suppression requires activation signals via the TCR; however, once activated their suppressor function is antigen non-specific [256]. Tregs exert their function by suppressing effector T cell responses, thus preventing the development of autoimmunity [257, 258]. Mice with a spontaneous mutation in the FoxP3 gene—known as ‘scurfy’ mice—develop fatal autoimmune diseases affecting multiple organs [254]. Similarly, humans with mutations affecting the FoxP3 gene suffer from severe autoimmunity (IPEX: immune polyendocrine enteropathy X-linked syndrome) [255]. In scurfy mice and IPEX patients, chronically activated self-reactive CD4^+^ T cells are found to be responsible for the development of pathology [259].

Naturally occurring Tregs (nTregs) develop in the thymus and constitute approximately 5–10 % of all peripheral CD4^+^ T cells in mice [260]. In mice, both CD25^high^ and CD25^low^ of the CD4^+^CD25^+^ population exhibit suppressor functions. However, in humans only CD4^+^CD25^high^ T cells are suppressive [261]. Human CD4^+^CD25^high^ Tregs account for 1–2 % of the total peripheral CD4^+^ T cell population [261]. It must also be considered that high levels of CD25 and FoxP3 are transiently expressed by recently activated human T cells, which do not display suppressor functions [262, 263]. Thus, a human Tregs-specific marker has yet to be identified. Despite this, markers important for the survival and the suppressive functions of Tregs have been identified such as CTLA-4 and glucocorticoid-induced TNF-receptor-related protein (GITR) [264, 265].

### Induced Tregs (iTregs)

Aside from nTregs, Tregs can also be induced from naïve CD4^+^ CD25^−^ T cells both in vitro and in vivo [266–268]. Both nTregs and iTregs do not proliferate in vitro and are able to suppress effector T cell responses both in vitro and in vivo [269]. Thus far, the differentiation of naïve T cells into iTregs in vivo has been documented in the murine intestine [177], inflamed lung [270], tumours [271] and transplanted tissues [272].

The possible underlying mechanisms of iTregs generation have also been explored in both humans and mice. In vitro generation of iTregs has been shown to be dependent on TGF-β and IL-2 [266, 273]. The VA metabolite RA also facilitates the generation of Tregs mediated by TGF-β following priming by DCs in the secondary lymphoid tissue [177, 180]. In addition, studies have shown that CD103^+^ DCs in the small intestinal LPs and the MLNs are capable of generating FoxP3^+^ Tregs in the presence of TGF-β and RA [177, 180]. ITregs can also be generated from CD4^+^CD25^−^ T cells cultured with rapamycin [274].

ITregs generated under different conditions may display different migratory patterns. iTregs generated in the presence of RA also express fully functional gut-homing receptors α_4_β_7_ and CCR9, therefore they migrate primarily to the gut [200]. ITregs generated in the presence of rapamycin express relatively high levels of CCR7 and possess a relatively greater lymphoid tissue-homing capacity [275]. Different characteristics of iTreg generated with different methods need to be taken into consideration when designing therapies for immune-mediated disorders.

### Treg homing to SLOs

Treg-mediated immune suppression operates in both SLOs [276] and non-lymphoid tissue [277, 278]. Appropriate trafficking and retention mediated by distinct chemokine and adhesion receptors are indispensable for Tregs to efficiently modulate immune response at sites where regulation is required [279–281]. nTregs express CD62L and CCR7, which facilitate their homing to the T cell area of LNs [276, 282, 283]. At the T cell zone, naïve Tregs may recognize antigens and become activated [284]. Upon activation, Tregs lose CCR7 expression and reduce CD62L expression [285, 286] and gain the expression of CXCR5, a chemokine receptor that promotes Treg migration into germinal centres in response to CXCL13, where they regulate the magnitude and characteristics of antibody responses [287, 288].

Migration to SLOs is required for Tregs to control the induction phase of an immune response [276, 289]. It was uncovered that CD62L^+^ Treg homing to LNs is necessary for the induction of transplantation tolerance [290]. CD62L^+^ Tregs, rather than CD62L^−^ Tregs, protect from acute graft-versus-host disease (GVHD) [291, 292] and efficiently delay diabetes in pre-diabetic NOD mice [293]. In contrast, Tregs from CCR7 knockout mice were inefficient in homing to LNs and did not suppress antigen-induced activation of T cell [294].

Within SLOs, Tregs need close physical contact with effector cells to exert their regulatory function [295]. Thus, effective suppression can only occur when homing of Tregs runs in parallel with that of effector T cells. Evidence suggests that Tregs and effector T cells home to similar areas of the LNs [289]. In a model of diabetes, in vivo two-photon microscopy revealed that Tregs co-localised with diabetogenic T cells in pancreatic LNs, and inhibited their antigen-dependent swarming and arrest [289]. Furthermore, using a recently described multiplex, quantitative imaging termed histocytometry, Liu et al. observed that within secondary lymphoid tissues in mice, Tregs exist in discrete clusters with IL-2-producing CD4^+^ T cells. Disruption of their physical co-clustering with effector T cells by inducible ablation of TCR expression by Tregs resulted in uncontrolled effector T cell responses [296].

### Treg cell homing to non-lymphoid tissue

Tregs need to migrate from circulation to non-lymphoid sites of inflammation to control effector immune responses [285, 297, 298]. Similarly to conventional T cells, Tregs in the microvasculature must undergo rolling, adhesion and transmigration mediated by distinct chemokine and adhesion receptors to arrive at the site of inflammation. Selectin ligands play a vital role for appropriate Tregs localisation in the periphery, particularly in the skin. In steady-state conditions, low-frequency rolling in dermal microvessels were P-selectin-dependent, whereas during most phases of inflammation, P-selectin and E-selectin both contributed to rolling [299]. Mice lacking PSGL-1, the main ligand required for P-selectin-dependent rolling, develop a more severe form of the EAE model of multiple sclerosis [300]. FucTVII, the enzyme responsible for generation of functional selectin ligands, is required for selectin-mediated leukocyte rolling in the microvasculature and leukocyte entry into the skin [301, 302]. Despite having normal in vitro regulatory capacity and populating lymphoid organs and peripheral sites such as the lung and intestine as efficiently as FucTVII sufficient Tregs, FucTVII deficient Tregs were severely restricted in their homing capacity to the skin, and unable to regulate contact hypersensitivity-associated skin inflammation [297].

Like conventional T cells, Treg trafficking to different tissues is determined by the combined expression of chemokine receptors and adhesion molecules [303]. Activated Tregs express α_E_β_7_, which binds its ligand E-cadherin and might predispose their retention to epithelial sites of inflammation in the periphery [285, 304]. Conversely, the α_E_^−^ Treg population was shown to be more efficient than α_E_^+^ Tregs at homing to LNs [297]. It has been shown that increased CLA or α_4_β_7_ expression by circulating human Tregs is associated with reduced risk of skin or gut acute GVHD, respectively [305]. Also like conventional T cells, the migratory properties of Tregs are shaped by the tissue microenvironment and organ-resident DCs during priming [198]. Tregs activated in the LNs draining a tissue acquire homing receptors that allow them to migrate back to the inflamed tissue. For example, Tregs activated in MLNs and PPs acquire α_4_β_7_ and CCR9, which direct their trafficking to the gut. By contrast, Tregs activated in PLNs express CCR8, which promote their migration to the skin [306].

Recent studies have shown that a large proportion of circulating human Tregs express the skin-homing receptors CLA and CCR4. In contrast, only a small number of circulating Tregs express gut-homing receptors α_4_β_7_ and CCR9 and α_E_β_7_ [307, 308]. This observation raises the question of how Tregs execute their suppressor functions in the gut [264]. A possible explanation is that MAdCAM-1, expressed constitutively on the postcapillary venules of intestine, is also capable of binding to CD62L found on Tregs in humans and mice [15, 297, 309]. CD62L therefore may target Tregs to the MLNs and the PPs where they carry out suppressor functions [307].

There is also evidence to suggest that Tregs can recirculate between SLOs and non-lymphoid tissues. A study by Tomura et al. showed that in steady-state Tregs constitutively migrated from the skin to draining LNs in mice. During skin inflammation, Tregs in circulation infiltrate the periphery, traffic to dLNs, and then recirculate back to the skin [310]. By doing so, Tregs can efficiently regulate both the induction phase and effector phase of immune response.

Treg migration to the inflamed liver has been shown to be supported by the common lymphatic endothelial and vascular endothelial receptor (CLEVER-1; also known as FEEL-1 and stabilin-1). Shetty and colleagues demonstrated increased endothelial expression of CLEVER-1 at sites of leukocyte recruitment to the inflamed human liver including sinusoids, septal vessels, and lymphoid follicles in inflammatory liver disease and tumor-associated vessels in hepatocellular carcinoma. Together with ICAM-1 and vascular adhesion protein-1 (VAP-1), CLEVER-1 can support transcellular migration of Tregs. CLEVER-1 inhibition reduced Treg transendothelial migration by 40 % and when combined with blockade of ICAM-1 and VAP-1 reduced it by >80 % [311].

The bone marrow is a critical site of immunity as studies have shown that long-lived, antibody-secreting plasma cells [312] and functional memory T cells [52, 53] reside in this organ. A subset of activated Tregs up-regulates CXCR4 expression and become responsive to SDF-1, enabling them to migrate to the bone marrow [313], where Tregs can suppress immune responses [53, 312, 314]. It has been described that in bone marrow of human and mice more than 25 % of CD4^+^ T cells are Tregs—as compared to 5–10 % of CD4^+^ T cells which are Tregs in the peripheral blood. Tregs exit from the bone marrow is achieved through granulocyte colony-stimulating factor (G-CSF) and reduced expression of SDF-1 [313]. Thus, it appears that Tregs are actively, rather than passively, recruited and retained in the bone marrow to regulate immune responses during homeostasis.

Long-term resident Tregs have been found in a wide range of tissues under homeostatic conditions, such as skin, gastrointestinal tract, lung, liver and adipose tissue [315–318]. Expression of particular sets of chemokine receptors not only is involved with Tregs recruitment to specific tissue sites, but also may facilitate retention of Tregs. Antigen recognition is also important in Treg survival and development of tissue tropic activity [319]. We and others have demonstrated that expression of TCR specific for an antigen expressed in a given tissue by Tregs promotes its recruitment, retention and clonal expansion in that location [320, 321]. nTregs predominantly recognise self-antigens [319]. We have reported that nTreg recognition of self-antigens expressed by endothelial cells in target tissue is instrumental for efficient Treg recruitment in vivo [320]. This may explain why recipient-derived Tregs are less efficient at modulating rejection of vascularized tissue graft [322]. A recent human study showed that Tregs comprise a high proportion (30–40 %) of CD4^+^ T cells in paediatric tissues but are present at much lower frequencies (1–10 %) in adult tissues. Paediatric tissue Treg cells suppress endogenous T cell proliferation and function, avoiding excessive activation as a result of diverse new antigens in early life [323]. Similarly, murine study by Scharschmidt et al. observed the accumulation of antigen-specific Tregs in neonatal skin is required to establish tolerance to skin commensals. Transiently blocking the migration of Tregs into the skin with S1PR1 antagonist FTY720 between postnatal days 5 and 11 to block the egress of lymphocytes from the thymus and skin dLNs [42] led to increased skin inflammation after challenge with same skin commensal bacteria in adult mice. This inflammation was associated with a reduction of antigen-specific Tregs and increased numbers of antigen-specific effector CD4^+^ T cells in the skin and skin dLNs [324]. The roles of tissue homing and antigen recognition are intertwined. Tregs utilize tissue homing molecules to migrate to the site of cognate antigen expression and lymphoid organs draining that site, and then receive TCR-derived signals that are critical to its survival and proliferation [325].

The number of Tregs in tissue has been observed to increase during inflammation. Regarding the relative contributions of tissue-resident versus recently recruited Tregs in the regulation of inflammation, it has been suggested that tissue-resident Tregs are sufficient to restrict mild inflammation stemming from recognition of commensal microbes or self-antigen in the periphery, but upon recognition of foreign microbial antigen, recruitment of additional Tregs is necessary to keep inflammation under control [315].

Tregs have been used to suppress allograft rejection in transplantation settings. T effector cells with high avidity for donor antigens migrate to an allograft to mediate transplant rejection [326]. Tregs must be able to migrate to transplanted organs to effectively suppress intra-graft anti-donor immune response. Intra-graft Tregs have been associated with tolerance induction. Tregs with unique chemokine profile have been shown to migrate to different graft tissues. CCR4 expression is associated with the recruitment of Tregs in heart allograft of tolerant mice [278]. Chemokine receptors CCR2, CCR4, CCR5 and P- and E-selectin ligands expression determines Tregs migration into islet allografts. CCR5 has been shown to play an important role in Tregs recruitment to both lymphoid tissues and GVHD target organs [327].

**Fig. 2:**
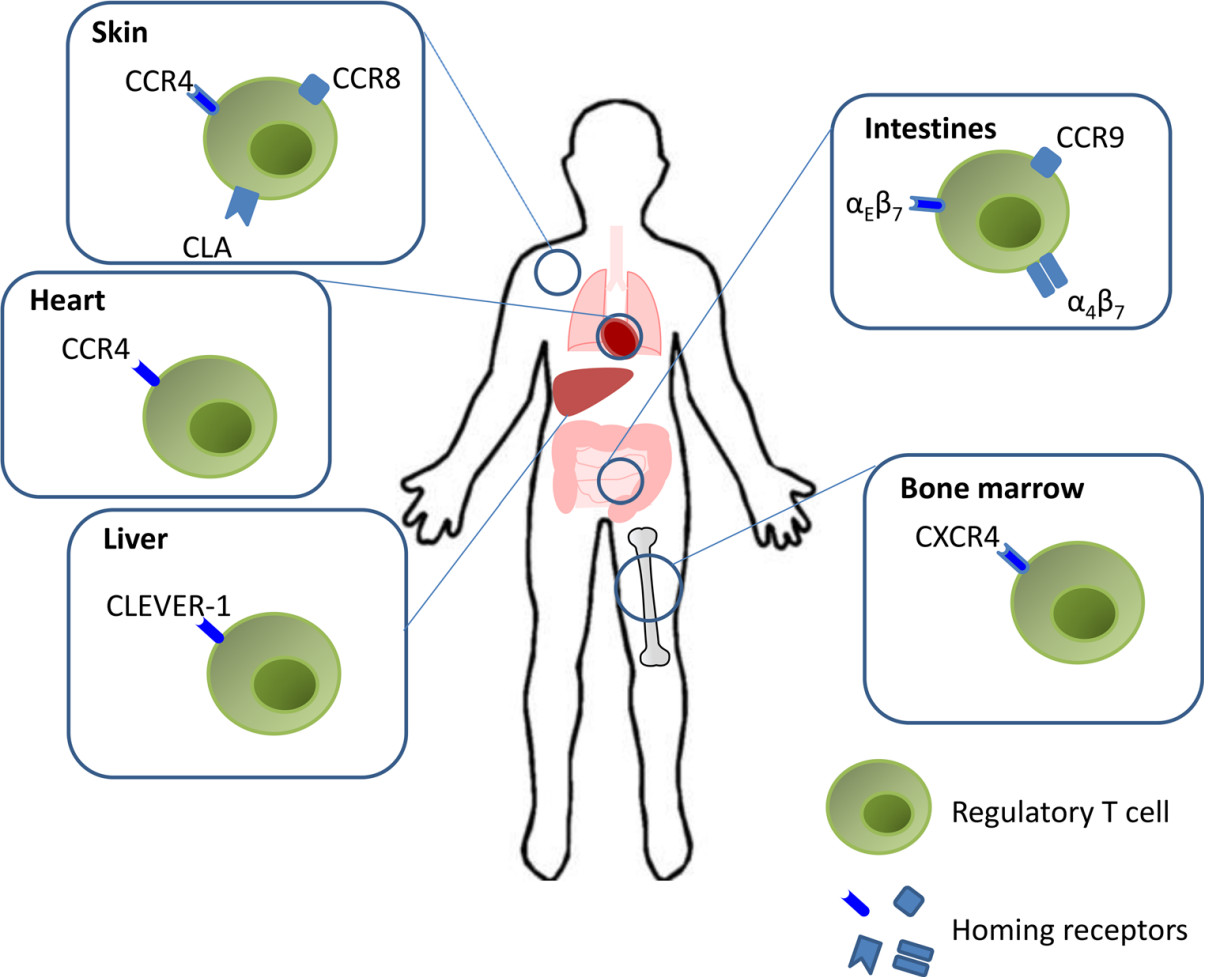
Regulatory T cell tissue homing. Modulation of immune responses by Tregs requires appropriate trafficking and retention. Homing signatures are mediated by distinct chemokine and adhesion molecules. Activated Tregs are predisposed to retention in the periphery due to the interaction of α_E_β_7_ with E-cadherin. Tregs expressing α_4_β_7_ and CCR9 migrate to the intestines. A subset of activated Tregs up-regulate CXCR4 expression and become responsive to SDF-1, enabling them to migrate to the bone marrow. Treg homing to the inflamed liver is mediated by interactions of the Tregs with hepatic sinusoidal endothelial cell expression of CLEVER-1. CCR4 expression is associated with the recruitment of Tregs in heart allograft of tolerant mice. Similar to effector T cells, Tregs express the skin-homing receptors CLA and CCR4 that bind to E-selectin and CCL17 respectively

A summary of the different homing pathways used by Treg cells is summarized in Fig. 2.

Emerging evidence suggests that Treg trafficking and compartmentalization can be modulated allowing efficient tissue targeting following ex vivo expansion. Siewert and colleagues have shown that Tregs can be configured both in vitro and in vivo with organ-selective homing properties allowing efficient migration into target tissue. For example, α_4_β_7_^+^ Tregs generated in vitro are able to effectively clear acute inflammation in the small intestine when transferred into the diseased animals [328]. This suggests that modulation of Tregs designed to serve for therapeutic purposes with specific homing properties is feasible for adoptive T cell therapy.

## Concluding remarks

While the majority of existing vaccines rely on circulating antibody for long-lasting protection, unique challenges posed by HIV or other sexually transmitted infections such as HSV require T cell-based vaccines or a combination of both T and B cell based vaccines. Successful T cell-based vaccination strategies depend not only on the generation of immunological memory against pathogen, but also on topographic memory of sites of infection as well as the establishment of T_RM_. Further understanding of how T cell tissue tropism is generated and maintained will aid in the design of efficacious vaccines. Due to the effect of local microenvironment on tissue homing acquisition, efforts should be made to design a vaccine that uses local immunization. Studies have shown that mucosal vaccination induces more recruitment of antigen-specific T cells to mucosal sites compared to distal immunization [329]. However, the recent finding that soluble factors can induce the acquisition of T cell homing receptors to selected tissue paves the way to the generation of combinatorial vaccines including ‘cues’ for the design of the desired tissue tropism.

A relatively unexplored issue is the plasticity of topographic memory. Understanding the genetic and epigenetic regulation of homing receptor expression will be crucial to the design of vaccination schedules involving combinatorial formulations. If, as it has been suggested, T cell trafficking programming is plastic, the possibility of reprogramming T cell tissue tropism upon a second antigen encounter provides great potential for a variety of therapeutic manipulations of immune responses.

## Abbreviations

ACD: Allergic contact dermatitis
Ang II: Angiotensin II
APCs: Antigen presenting cells
BATF: Basic leucine zipper transcription factor
CLA: Cutaneous lymphocyte-associated antigen
CNS: Central nervous system
CTLA-4: Cytotoxic T lymphocytes antigen 4
DCs: Dendritic cells
DETCs: Dendritic epidermal T cells
EAE: Experimental autoimmune encephalomyelitis
ECs: Endothelial cells
FoxP3: Forkhead Box P3
GALT: Gut-associated lymphoid tissue
GPR: G protein-coupled receptor
GVHD: Graft-versus-host disease
HGF: Hepatocyte growth factor
HIV: Human immunodeficiency virus
HSV: Herpes simplex virus
IELs: Intraepithelial lymphocytes
IPEX: Immune polyendocrine enteropathy X-linked syndrome
KLF2: Krüppel-like factor 2
LFA: Lymphocyte function-associated antigen
LNs: Lymph nodes
LP: Lamina propria
MAdCAM-1: Mucosal addressin cell adhesion molecule-1
MHC: Major histocompatibility complex
MLCs: Memory lymphocyte clusters
MLNs: Mesenteric lymph nodes
MMP: Matrix metalloproteinases
mTOR: Mammalian target of rapamycin
NOD: Non-obese diabetic
PLNs: Peripheral lymph nodes
PI3K: Phosphatidylinositol-3′-kinase
PNAd: Peripheral lymph node addressin
PSGL-1: P-selectin glycoprotein ligand-1
PPs: Peyer’s patches
RA: Retinoic acid
RAR: Retinoic acid receptor
RALDH: Retinaldehyde dehydrogenase
S1PR1: Sphingosine-1-phosphate receptor 1
S1P: Sphingosine-1 phosphate
SDF-1: Stromal-derived factor-1
SLOs: Secondary lymphoid organs
TEM: Trans-endothelial migration
TGF-β: Transforming growth factor beta
T_CM_: Central memory T cells
T_EFF_: Effector T cells
T_EM_: Effector memory T cells
T_RM_: Tissue resident memory T cells
Th: T helper cells
Tregs: Regulatory T cells
iTregs: Induced Tregs
nTregs: Naturally occurring Tregs
VA: Vitamin A
VCAM-1: Vascular cell adhesion molecule-1
VLA: Very late antigen (VLA)
